# *N*-Myristoyltransferase from *Leishmania donovani*: Structural and Functional Characterisation of a Potential Drug Target for Visceral Leishmaniasis

**DOI:** 10.1016/j.jmb.2009.12.032

**Published:** 2010-03-05

**Authors:** James A. Brannigan, Barbara A. Smith, Zhiyong Yu, Andrzej M. Brzozowski, Michael R. Hodgkinson, Asher Maroof, Helen P. Price, Franziska Meier, Robin J. Leatherbarrow, Edward W. Tate, Deborah F. Smith, Anthony J. Wilkinson

**Affiliations:** 1 York Structural Biology Laboratory, Department of Chemistry, University of York, York YO10 5YW, UK; 2 Centre for Immunology and Infection, Department of Biology/Hull York Medical School, University of York, York YO10 5YW, UK; 3 Department of Chemistry, Imperial College, London SW7 2AZ, UK

**Keywords:** ARF, ADP-ribosylation factor, DIG, digoxigenin, HASP, hydrophilic acylated surface protein, *HYG*, hygromycin, NEO, neomycin, NHM, non-hydrolysable myristoyl-CoA analogue, NMT, *N*-myristoyltransferase, ORF, open reading frame, *PAC*, puromycin, SPA, scintillation proximity assay, VL, visceral leishmaniasis, CaNMT, HsNMT, LdNMT and ScNMT, *N*-myristoyltransferase from *Candida albicans*, *Homo sapiens*, *Leishmania donovani* and *Saccharomyces cerevisiae*, respectively, *N*-myristoyltransferase, *Leishmania*, visceral leishmaniasis, crystal structure, drug target

## Abstract

*N*-Myristoyltransferase (NMT) catalyses the attachment of the 14-carbon saturated fatty acid, myristate, to the amino-terminal glycine residue of a subset of eukaryotic proteins that function in multiple cellular processes, including vesicular protein trafficking and signal transduction. In these pathways, N-myristoylation facilitates association of substrate proteins with membranes or the hydrophobic domains of other partner peptides. NMT function is essential for viability in all cell types tested to date, demonstrating that this enzyme has potential as a target for drug development. Here, we provide genetic evidence that NMT is likely to be essential for viability in insect stages of the pathogenic protozoan parasite, *Leishmania donovani*, causative agent of the tropical infectious disease, visceral leishmaniasis. The open reading frame of *L. donovaniNMT* has been amplified and used to overproduce active recombinant enzyme in *Escherichia coli*, as demonstrated by gel mobility shift assays of ligand binding and peptide-myristoylation activity in scintillation proximity assays. The purified protein has been crystallized in complex with the non-hydrolysable substrate analogue *S*-(2-oxo)pentadecyl-CoA, and its structure was solved by molecular replacement at 1.4 Å resolution. The structure has as its defining feature a 14-stranded twisted β-sheet on which helices are packed so as to form an extended and curved substrate-binding groove running across two protein lobes. The fatty acyl-CoA is largely buried in the N-terminal lobe, its binding leading to the loosening of a flap, which in unliganded NMT structures, occludes the protein substrate binding site in the carboxy-terminal lobe. These studies validate *L. donovani* NMT as a potential target for development of new therapeutic agents against visceral leishmaniasis.

## Introduction

The leishmaniases, caused by species of the kinetoplastid parasite *Leishmania*, are a spectrum of diseases associated with immune dysfunction that give rise to > 2 million new cases each year in 88 countries, with 367 million people at risk.^1,2^ Clinical symptoms range from the disfiguring skin lesions of cutaneous leishmaniasis to the often fatal visceral leishmaniasis (VL). These are exacerbated in children and immunocompromised patients, such as those diagnosed as HIV positive. There is a limited range of effective drugs in use including Pentostam, amphotericin B (in its liposomal formulation, AmBisome) and miltefosine, with a few others in clinical trials.^3^ However, delivery regimens can be complex, while miltefosine, the only oral VL drug currently available, is teratogenic, limiting its distribution in endemic populations. Moreover, resistance is an increasing problem and there are no effective vaccines currently available.^4^ There is therefore a pressing need for new, preferably orally administered drugs, particularly for VL.^2^ Previous studies have identified myristoyl-CoA–protein *N*-myristoyltransferase (NMT) as a suitable candidate for drug development against protozoan parasitic infections, including *Leishmania major*, the causative agent of cutaneous leishmaniasis, as well as *Plasmodium falciparum* and *Trypanosoma brucei*, causative agents of human malaria and African sleeping sickness, respectively.^5–9^

NMT is ubiquitous in eukaryotic cells in which it catalyses the co-translational addition of the C_14:0_ fatty acid, myristate, via an amide bond, to the N-terminal glycine residue of a subset of proteins. N-Myristoylation plays a role in targeting proteins to membrane locations, mediating protein–protein interactions and stabilizing protein structures (reviewed in Ref. 10). N-Myristoylation by NMT proceeds via an ordered Bi–Bi reaction mechanism; binding of myristoyl-CoA opens up a second pocket for docking of the substrate protein.^11,12^ The myristate group is then transferred to the N-terminal glycine of the substrate in a nucleophilic addition–elimination reaction, followed by stepwise release of first the free CoA and then the *N*-myristoylated protein.^11,13^

NMTs have been well-characterised in *Saccharomyces cerevisiae*^14^ and human cells^15^ and are essential for viability in pathogenic fungi.^16^ Comparative sequence and biochemical analyses have demonstrated high conservation of the myristoyl-CoA binding sites in human and fungal NMTs but divergent peptide-binding specificities.^17^ Given these observations, peptide-based and peptidomimetic inhibitors have been developed that show selectivity against the NMTs of fungal species as compared to human NMT.^18–20^ As a consequence, NMT has been the target of a number of antifungal drug development programmes, with the focus on selective inhibitors acting at the peptide-binding pocket. In the preliminary stages of commercial development of fungal NMT inhibitors, high selectivity and specificity were achieved using two different core scaffolds: benzothiazoles (Pfizer, unpublished data) and benzofurans (Roche).^21^ Further progress was curtailed, however, due to fungal species specificity of the best leads—new antifungals of higher efficacy are required to be broad spectrum, thereby reducing the necessity for clinical diagnosis prior to treatment.

This rationale does not apply to parasitic diseases such as leishmaniases, which are readily diagnosed according to clinical, molecular and epidemiological indicators. In *L. major*, the single-copy *NMT* gene is constitutively expressed in all parasite stages,^7^ with the 421-residue (48.5 kDa) monomeric protein localizing to both cytoplasmic and membrane fractions. Genome sequence analysis has identified 62 putative protein substrates for N-myristoylation in this organism, of which ∼ 10 have been identified by metabolic labeling.^7,22^ These include ADP-ribosylation factors (ARFs) and the infective stage-specific hydrophilic acylated surface proteins (HASPs). HASPB, which requires both N-myristoylation and palmitoylation for transport to the parasite plasma membrane,^23^ is a vaccine candidate for VL.^24–26^ The *L. major* NMT has been genetically validated as essential for the viability of insect-stage promastigotes using targeted gene disruption techniques.^7^

Here, we extend our studies on NMT to *Leishmania donovani* (LdNMT), the principal causative agent of the most serious form of leishmaniasis, and demonstrate that the *L. donovani NMT* gene is also likely to be essential for viability in extracellular parasites. With the aim of exploiting LdNMT for drug discovery, we have overproduced and purified the recombinant protein in *Escherichia coli* and characterised its ligand-binding properties and enzyme activity. In addition, we have determined by molecular replacement the high-resolution crystal structure of LdNMT in complex with a non-hydrolysable myristoyl-CoA analogue. The structure is discussed in relation to sequence alignments and the structures of unliganded, binary and ternary complexes of yeast and fungal NMTs.

## Results and Discussion

### The *L. donovani NMT* gene is essential for the viability of insect-stage parasites

**Fig. 1:**
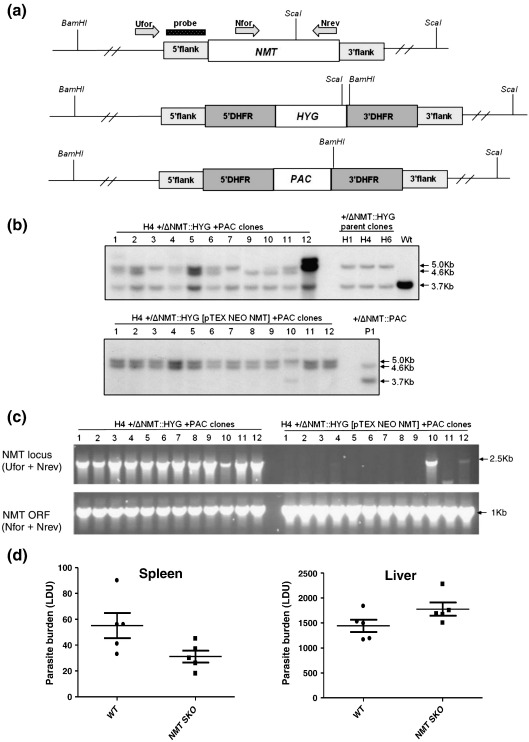
The *NMT* gene codes for an essential enzyme in *L. donovani* promastigotes. (a) Restriction maps of the wild-type *NMT* locus and targeted single alleles containing replacement by either the *HYG* or *PAC* genes. 5′Flank and 3′flank boxes represent *NMT* flanking regions used for gene targeting. 5′DHFR and 3′DHFR boxes represent 5′ and 3′ dihydrofolate reductase flanking regions. Arrows show positions of primers used in PCR analysis of clones in (c). Position of the DIG-labeled probe hybridized to membranes in (b) indicated by stippled box. (b) Southern blot analysis of ScaI and BamHI digests of parasite genomic DNA hybridized with 5′U probe, which hybridizes to the 5′ flanking region. Arrows indicate the following bands: 5.0 kb, *HYG* replacement at *NMT* locus; 4.6 kb, *PAC* replacement at *NMT* locus; 3.7 kb, Wt *NMT* locus. Upper panel: wild type (Wt), three single allele *HYG* replacement (+/ΔNMT::*HYG* H1, H4, H16) and 11 clones generated from H4 +/ΔNMT::*HYG* parent clone by attempted replacement with *PAC* (H4 +/ΔNMT::*HYG* + *PAC* clones 1–12). Lower panel: 12 clones generated from an H4 +/ΔNMT::*HYG* parent clone overexpressing NMT by attempted replacement with *PAC* (+/ΔNMT::*HYG* [pTEX NEO NMT] +  *PAC*) and a single allele *PAC* replacement (+/ΔNMT::*PAC*, P1). (c) PCR analysis of the wild-type NMT locus (using primers Ufor and Nrev) and NMT ORF (using primers Nfor and Nrev). Clones are as described in (b). (d) Groups of five BALB/c mice were inoculated with 4 × 10^7^*L. donovani* metacyclic promastigotes (either wild-type parasites or one of the +/ΔNMT::*HYG* clones [NMT SKO]) intravenously. Parasite burdens in the spleen (left panel) and liver (right panel) were determined at 28 days post-infection by examination of methanol-fixed, Giemsa-stained tissue imprints, as previously described ^25^. Data are presented as Leishman Donovan units (LDU), in which LDU represents the number of intracellular amastigotes/1000 host cell nuclei  ×  organ weight (mg). Statistical analysis was performed by a paired Student's *t*-test.

In *L. major*, targeted gene deletion methods were used to demonstrate that the single–copy *NMT* gene is essential for viability in promastigotes.^7^ Here, the same approach has been taken to investigate whether *L. donovani NMT* gene function is also an essential requirement for propagation of this parasite species. Linear constructs were generated, carrying drug resistance marker genes [*HYG* (hygromycin resistance gene) or *PAC* (puromycin resistance gene)] flanked by dihydrofolate reductase sequences conferring constitutive expression. These were targeted to the *NMT* locus, using 5′ and 3′ intergenic regions for homologous recombination (Fig. 1a). Numerous heterozygous parasite clones (+/ΔNMT::*HYG* or +/ΔNMT::*PAC*) containing an integrated copy of one of the drug resistance genes were obtained using this approach. Three +/ΔNMT::*HYG* clones (H1, H4 and H16) were independently transfected with pTEX NEO (neomycin) NMT^7^ to generate clones overexpressing NMT from the pTEX plasmid (H1, H4, H16 +/ΔNMT::*HYG* [pTEX NEO NMT]). Attempts were then made to replace the second NMT allele in all six clones by transfection with the *PAC* linear targeting construct. Twelve clones from each transfection were isolated (a total of 72 transgenic events) and further analysed by PCR, DNA blotting and immunoblotting. The results of these analyses are summarized in Table 1 and in Fig. 1, where examples of Southern blotting and PCR analyses for clones derived from *PAC* targeting of the +/ΔNMT::*HYG* parent clone H4 are shown. Eleven out of 11 of these clones have retained a wild-type NMT allele as shown by DNA blotting (Fig. 1b, upper panel) and 12 of 12 as analysed by PCR amplification (c, lower panel). By contrast, the majority (10/12) of the *PAC* targeted NMT overexpressing clones have lost the wild-type NMT allele, with only clone 10 showing retention of this gene by Southern blotting (b, lower panel) and clones 10 and 12 by PCR amplification (c, upper panel).

Of the 72 clones analysed, none demonstrated a complete absence of NMT coding sequence (see Table 1 for the cumulative results from this analysis). Many of the *PAC*-targeted +/ΔNMT::*HYG* H4 clones showed evidence of both *HYG* and *PAC* replacement at the NMT locus in addition to the presence of a wild-type NMT gene elsewhere in the genome (e.g., clones 1, 2, 5, 6 and 11; Fig. 1b, upper panel). However, the majority of NMT overexpressing clones had lost both wild-type NMT alleles by gene replacement, while retaining a copy of the NMT gene, presumably episomally. Therefore, in these studies, we were unable to generate a transgenic parasite line null for NMT expression. Our overall interpretation of these data is that *L. donovani* promastigotes require at least one allele of the *NMT* gene for successful propagation in culture. Expression of ∼ 50% of the wild-type level of LdNMT is also sufficient for parasite survival following phagocytosis by macrophages *in vivo*, as two +/ΔNMT::*HYG* clones were successfully passaged through immunodeficient Rag^−/−^γ^−/−^ mice.^27^ Similarly, intravenous inoculation of susceptible BALB/c mice with wild-type *L. donovani* and one of the +/ΔNMT::*HYG* clones (heterozygous for NMT) revealed similar parasite burdens in the liver and spleen at 28 days post-infection (Fig. 1d). There was no significant difference between wild-type and heterozygous infections in either tissue at this time when insect-stage promastigotes have differentiated into intracellular amastigotes. It is not currently possible to selectively knock down gene expression in intracellular *Leishmania* and test at what point parasite viability is compromised with decreasing expression and activity of LdNMT.

### Characterisation of NMT from *L. donovani*

We have cloned and sequenced the gene encoding LdNMT from *L. donovani* strain LV9. Relative to the coding sequence of the *NMT* gene of the closely related *Leishmania infantum* strain JPCM5,^28^ there are just two nucleotide differences (C to T transitions at positions 258 and 492) neither resulting in an amino acid substitution. There are only 11 amino acid differences between LdNMT and the NMT of the *L. major* Friedlin strain.

**Fig. 2:**
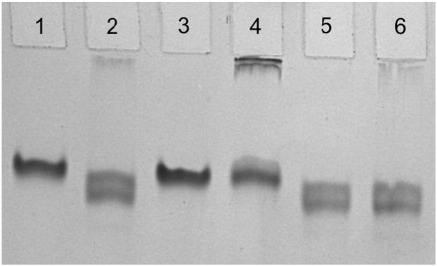
Protein mobility shifts on native electrophoresis. Altered mobility of LdNMT protein on non-denaturing PAGE (7.5% polyacrylamide; run conditions, 90 V for 100 min) corresponds to complex formation with cofactor. 3 μg of protein (lane 1) was incubated with myristoyl-CoA in 10-fold molar excess (lane 2) or 2-fold excess of ARF peptide (lane 3). Low-mobility material is visible when both cofactor and peptide are present (lane 4), which does not appear when the non-hydrolysable cofactor NHM is used (lane 5, protein + NHM; lane 6, +NHM + peptide).

The *L. donovani* NMT was overproduced in *E. coli* and purified in good yield (5 mg l^− 1^ of cell culture; see Materials and Methods). The protein migrates as a single band in nondenaturing gels following electrophoresis (Fig. 2, lane 1). For ligand binding and activity assays, we used a peptide derivative representing a canonical myristoylation motif derived from ARF and the non-hydrolysable myristoyl-CoA analogue *S*-(2-oxo)pentadecyl-CoA (NHM) prepared by an alternative synthetic route as described in Materials and Methods and Supplementary Material. As shown in Fig. 2, the mobility of the isolated protein (lane 1) increases following incubation with either myristoyl-CoA (lane 2) or NHM (lane 5) consistent with the formation of binary complexes of LdNMT and the fatty acyl-CoA species. Incubation with peptide alone (lane 3) has no influence on mobility, suggesting that LdNMT–peptide complexes are not being formed, consistent with observations on orthologous NMTs that there is compulsory order in substrate binding and that the peptide binding site is not accessible prior to fatty acyl-CoA binding. Following preincubation with myristoyl-CoA and addition of peptide substrate, there is additional staining of material with low gel mobility (lane 4) consistent with chemical transformation and the formation of myristoylated peptides, which are not formed when NHM is used as cofactor (lane 6).

The kinetics of the LdNMT-catalysed reaction were measured by a scintillation proximity assay (SPA) using [^3^H]myristoyl-CoA and the C-terminally biotinylated ARF peptide GLYVSRLFNRLFQKK(Biotin)-NH_2._^5^ The *K*_m_^app^ values of LdNMT for myristoyl-CoA and the peptide are 17.5  ±  2.9 nM and 0.23  ±  0.12 μM, respectively (Table 2). These values cannot be compared directly with those measured for the NMTs of other species as the assay substrates and conditions are different; nevertheless, the numbers measured are of the expected order of magnitude.^29,30^ NHM was shown to be a potent inhibitor of the LdNMT-catalysed transfer of myristate to the peptide. The IC_50_ value of 68.7 ± 4.2 nM (see Supplementary Material) is in the range reported for the NMTs from other species.^17,31^

### Crystal structure solution and model

The structure of LdNMT was solved by molecular replacement using the coordinate set 1NMT.pdb^32^ as the search model. The refined coordinates have good agreement with the X-ray data extending to 1.42 Å resolution, with *R*_cryst_ and *R*_free_ values of 14.6% and 19.1%, respectively (Table 3). There is a single LdNMT molecule in the asymmetric unit and the chain is well defined in the electron density maps, with the exception of residues 1–10 at the amino terminus and residues 83–85 and 334–339, which are not defined by the maps and assumed to be disordered. The model contains one molecule of *S*-(2-oxo)pentadecyl-CoA and 378 water molecules.

**Fig. 3:**
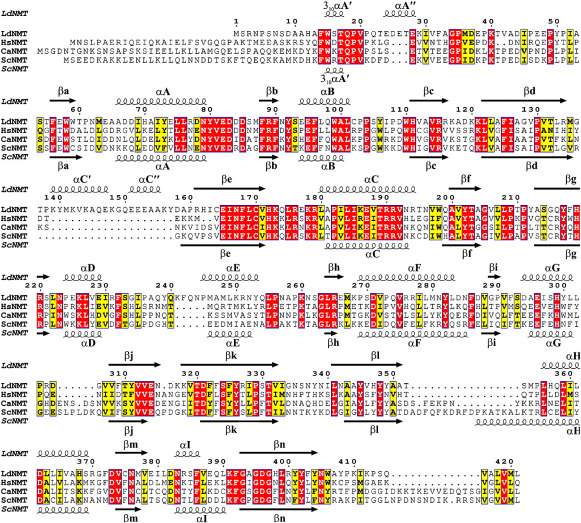
Sequence alignment of NMTs. Structure-based amino acid sequence alignment of NMT proteins of known structure (Ld, *L. donovani*; Hs, human; Ca, *C. albicans*; Sc, *S. cerevisiae*). Strictly conserved residues are coloured red and well-conserved residues are coloured yellow. The secondary-structure elements of LdNMT and ScNMT are shown above and below the sequence alignment, respectively. This figure was generated using ESPript.^34^

In describing the structure, the secondary-structure nomenclature used by earlier workers will be adopted^32,33^ in which helices are denoted with uppercase letters and strands with lowercase letters (Fig. 3). Secondary-structure elements additional to those observed in the *C. albicans* NMT (CaNMT) are denoted by one or more primes (′). The mean atomic temperature factor for main-chain atoms is low at 15 Å^2^. The regions corresponding to A″, the C′C″ helical pair, the eC loop, the DEhF element, and loops Ab and kl (which contain missing residues), have residues with significantly higher average main-chain temperature factors, suggesting that these regions of the structure have independent mobility. As described below, some of these regions are of functional importance.

### Overall structure

**Fig. 4:**
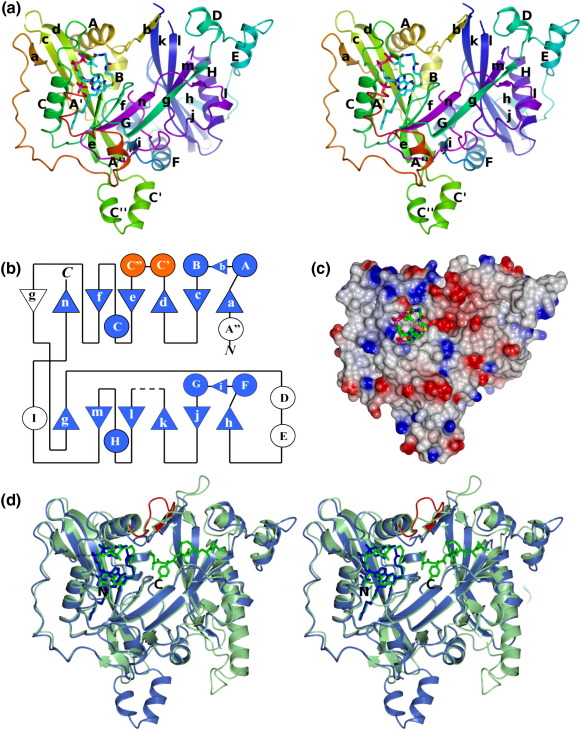
The three-dimensional structure of LdNMT. (a) Stereo ribbon representation of LdNMT in its complex with NHM, which is shown in cylinder format and coloured by atom type: carbon, cyan; oxygen, red; nitrogen, blue, sulfur, yellow; phosphorus, magenta. The protein chain is colour ramped from residue 11 at the N-terminus (red) to residue 421 at the C-terminus (magenta). The secondary-structure elements are labelled with uppercase letters for α-helices and lowercase letters for β-strands. The ' and ” superscripts indicate elements additional to those observed in the first NMT structures to be described. (b) Topology diagram of LdNMT with secondary-structure elements labelled and with α-helices represented as circles and β-strands as triangles. Lobe 1 is shown above lobe 2 with the light blue shading indicating the tandemly duplicated region. The italicised N and C indicate the chain termini. The additional helices C′ and C ″ appear as an insert coloured in orange in lobe 1 at a region that is disordered in lobe II. (c) Electrostatic surface representation of LdNMT. The molecule is shown in an orientation similar to that in (a). Blue and red surface colouring represents positive and negative electrostatic potential, respectively. The NHM ligand is shown in cylinder format and coloured by element as above except that carbon atoms are in green. (d) Comparison of the structures of LdNMT and ScNMT. The structures of LdNMT–NHM and ScNMT–NHM–GLYASKLA (PDB code 1IIC^35^) were superposed using the SSM superpose routine in CCP4MG. The structures are displayed as ribbons, LdNMT (blue) and ScNMT (green), with the ligands represented in cylinder format. The Ab loop from the superposed ScNMT–myristoyl-CoA binary complex (1IIC) is shown in red.

The core of the structure is a 12-stranded β-sheet that is strikingly twisted with strand order acdefngmlkjh (Fig. 4a). The β-sheet forms an open barrel that together with the three α-helices A, B and G encloses a volume occupied by the C-terminal segment of the polypeptide, so that the α-carboxylate of the polypeptide chain is at the heart of the molecule. The molecule has two lobes, each of which has a multistranded β-sheet element at its core flanked by two α-helices on one face and a single α-helix on the other face (Fig. 4a). For the N-terminal lobe, the sheet is made up of strands acdefng with helices A and B packed onto one face and helix C onto the other. For the C-terminal lobe, the sheet comprises strands gmlkjh against one face of which helices F and G are packed, with helix H packed onto the other face.

There is pseudosymmetry within the molecule in the form of tandem duplication such that the topology of residues 56–134 and 161–221 matches that of residues 264–404. The repeated segment has a βαβαβββαββ topology (Fig. 4b) that largely defines the two lobes with the exception of the final β-strands, g and n, which cross over into the opposite lobe. Residues 135–160 represent a prominent insertion between strands d and e in the sequence of the N-terminal lobe, and these residues form a helix–loop–helix containing C′ and C″, which protrudes from the base of the molecule (Fig. 4a). The corresponding segment of the C-terminal lobe between strands k and l comprises just eight residues most of which are disordered (Fig. 4b).

Outside the region of duplication, residues 221–264, containing helices D and E and associated with lobe 2, form the right flank of the molecule when viewed as in Fig. 4a. The left flank comprises residues 11 to 56 at the amino terminus of the polypeptide, which form a long meandering segment that, with the exception of a short 3_10_ helix (A′) at the start of the chain and a six-residue α-helical segment (A″), lacks secondary structure. These largely exposed residues are often disordered in NMT structures. Residues 404–421 at the carboxyl terminus also lack secondary structure, though here the last six residues are threaded through into the centre of the molecule (Fig. 4a).

The structure of LdNMT is very similar to that of the *S. cerevisiae* enzyme (41% sequence identity, rmsΔ = 1.4 Å for 373 equivalent Cα atoms). In NMT from *S. cerevisiae* (ScNMT), the A′ 3_10_ helix is present but residues corresponding to the A″ α-helix in LdNMT are in an extended conformation (Figs. 3 and 4c). The yeast NMT has an extra 26 residues at the N-terminus relative to LdNMT, though these appear to be disordered. The most striking distinguishing feature of LdNMT is the additional pair of α-helices C′ and C″ (Fig. 4c). These structural elements correspond to a prominent ∼ 20-residue insertion at residue 140 in the sequence of the *Leishmania* NMTs (Fig. 3). This helix pair is clearly visible at the bottom of Fig. 4c, protruding from the main body of the protein. Elsewhere, helix H in LdNMT has three fewer turns than the corresponding helix in ScNMT (Fig. 4c) and the lH segment is shorter, these differences accounting for the gap in the alignment after residue 350 (Fig. 3). The LdNMT also has a much shorter segment connecting the final β-strand n with the C-terminus. These differences in sequence/structure are on the face of the β-sheet distal to the active site and substrate-binding cleft and therefore unlikely to have functional significance.

### Substrate binding and loop movements

Detailed studies of NMTs have led to the proposal of a structurally informed mechanism of action for ScNMT,^35^ which may be extrapolated to LdNMT, in which firstly the myristoyl-CoA and secondly the target protein substrates bind to the enzyme. The terminal carboxylate group of Leu421 acts as a base to deprotonate the α-amino group of the terminal glycine residue of the substrate, activating it for nucleophilic attack on the carbonyl carbon of the thioester linkage in myristoyl-CoA. It appears that a conformational change following binding is required to bring the reacting groups into appropriate juxtaposition for the chemistry to proceed. The charge in the transition state of the reaction is dissipated through the carbonyl oxygen, the oxyanion, with stabilizing interactions with the amide groups of Phe168 and Leu169. Following acyl group transfer, there is ordered dissociation of first the CoA and finally the myristoylated peptide.

The ordered binding of substrates is accounted for by the displacement upon myristoyl-CoA binding of the Ab loop, which partially occludes the binding site for the N-terminal peptide of the substrate protein.^33,35^ This loop, which serves as a lid that opens and closes over the catalytic site, has variable conformations among the different structures. It partially occludes the myristoyl-CoA and peptide binding sites in the structure of the uncomplexed CaNMT, while in the binary and ternary complexes of ScNMT the loop has swung upward to different extents, opening up the active site. In this way, the binding of myristoyl-CoA assists peptide binding by ensuring the lid is open and the second substrate can bind unhindered. The central residues of the lid in LdNMT are missing from the model; moreover, the flanking residues of the Ab loop have much higher than average temperature factors, suggesting intrinsic mobility of this segment of the structure. There are some clashes between residues of this loop and the GLYASKLA peptide from ScNMT following superposition of the protein backbones (see below). Taken together, it seems that the Ab loop is closed in the uncomplexed enzyme and that following fatty acyl-CoA binding the Ab loop adopts an ensemble of structures that facilitate the binding of the N-terminal peptide of the substrate protein.

### Fatty acyl-CoA binding

**Fig. 5:**
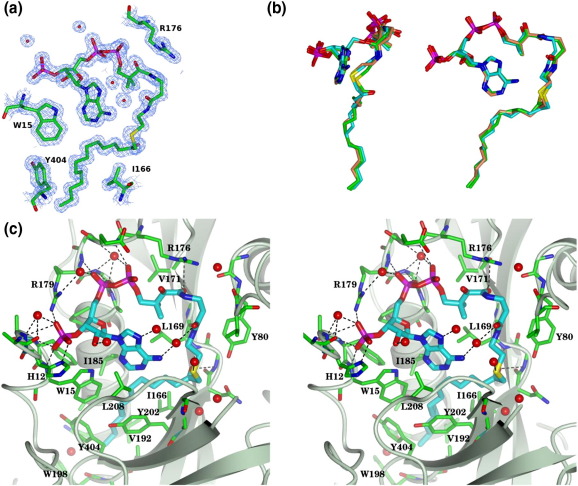
Fatty acyl-CoA binding to LdNMT. (a) 2*F*_o_ − *F*_c_ electron density contoured at 3σ and displayed in the neighbourhood of the NHM species in the refined LdNMT model. (b) Orthogonal views of the fatty acyl-CoA in the LdNMT binary complex and ScNMT binary and ternary complexes. Carbon atoms are coloured according to structure: LdNMT–NHM, cyan; ScNMT–myristoyl-CoA, green; ScNMT–NHM–GLYASKLA, coral. The fatty acyl-CoA species were superposed by least-squares methods. (c) Stereo view of the NHM and neighbouring residues in LdNMT. The fatty acyl-CoA is shown with thicker bonds relative to the protein and its carbon atoms are coloured in cyan rather than green. The remaining atoms are coloured as follows: oxygen, red; nitrogen, blue, sulfur, yellow; phosphorus, magenta. The backbone of the protein is indicated as a light blue ribbon. Polar interactions between the protein and the fatty acyl-CoA are indicated by dashed lines. The figure was prepared with CCP4MG.^36^

LdNMT was co-crystallised with NHM, a non-hydrolysable analogue of myristoyl-CoA with a methylene group intervening between the sulfur and the carbonyl carbon in what would otherwise be the thioester linkage in myristoyl-CoA. As shown in Fig. 5a, all 64 heavy atoms of the NHM are clearly defined in the electron density maps with this ligand residing in one side of a prominent groove running across the molecule (Fig. 4c). The fatty acyl-CoA binds to the N-terminal lobe in a site that is formed by the eCf elements that encircle the cofactor, with the A′ 3_10_ helix and the Ab loop forming lateral interactions (Figs. 4a and 5c). In its bound conformation, 1070 Å^2^ or 88% of the cofactor's solvent-accessible surface area of 1215 Å^2^ is buried by interaction with the protein. The extensive interaction surface is consistent with the low IC_50_ of NHM.

In the complex, NHM takes up a conformation that has been described elsewhere^33^ as resembling a question mark, with the extended pantetheine and fatty acyl species folding around the adenine base. As a result, the majority of the interactions of the adenine base and the ribose sugar are intramolecular, with interactions with the protein confined to apolar contacts with Trp15 and Leu208 (Fig. 5c). In contrast, the 3′ phosphate group forms a multitude of interactions with the protein including ion pairing with the side chains of His12 and Arg179 and charge–dipole interactions with the main-chain amides of residues 14 and 15 and water-mediated interactions with the carbonyl and amide of residues Ala11 and Ala13, respectively. The 5′ diphosphate is stabilized by direct interactions with the main-chain amides of Glu177 and Arg179 and water-mediated interactions with the main-chain amide of Leu180 and the guanidine group of Arg179. There are polar contacts between O9 of the pantetheine (O9p) and the amide group of Val171 and between N4p and the carbonyl group of Leu169. A further water-mediated interaction occurs between O6p of pantetheine and the exocyclic amino group of the adenine base (Fig. 5c). Further apolar contacts of the pantetheine are formed with Tyr80, Val81, Val171, Ala181 and Arg176.

The carbonyl group of the pentadecyl moiety is directed into the protein interior to form hydrogen bonds with the main-chain amide groups of Phe168 and Leu169, these groups forming the oxyanion hole. The aliphatic chain of the pentadecyl species is in a largely extended conformation with slight kinking of the chain at C5–C6 and C8–C9. It runs into a deep hydrophobic pocket lined by residues Trp15, Ile166, Leu169, Ile185, Val192, Trp198, Gln199, Ala200, Tyr202, Leu208, and Tyr404 (Fig. 5). The distal portion of the aliphatic chain runs antiparallel to helix C and parallel to strand f with the loop connecting these elements of secondary structure forming the base of the pocket.

The conformation and interactions of the fatty acyl-CoA in LdNMT are well conserved in the structures of the yeast and fungal NMTs.^35,37^ As shown in Fig. 5b, the NHM molecule from the complex reported here is superimposed on the fatty acyl-CoA entities from (i) the ScNMT complex with NHM and the octapeptide GLYASKLA [Protein Data Bank (PDB) entry 1IID] and (ii) the ScNMT complex with myristoyl-CoA (PDB entry 1IIC). The rmsΔ following least-squares minimization of the positions of equivalent atoms is ∼ 0.6 Å in each case. The closest overlap (0.3 Å) is at the active centre where the carbonyl oxygen of the fatty acid is directed into the oxyanion hole. The sulfur atom of the NHM is displaced by just 1.1 Å from its location in myristoyl-CoA, despite the presence of the methylene group between the carbonyl group and the sulfur in the former.

### Peptide binding site in LdNMT and HsNMT and implications for drug design

Alignment of the sequence of LdNMT and human NMT (HsNMT) reveals 42% residue identity over their 421 and 495 residues, respectively. We are interested here in the sequence differences between the *L. donovani* and human enzymes and their structural consequences for the discovery of selective inhibitors of LdNMT. The myristoyl-CoA binding site in NMTs is very well conserved among species, and the interactions of fatty acyl-CoA with the enzyme are conserved in the structures that have been determined. Attention has therefore been directed toward the development of inhibitors that bind in the peptide binding site. There is conservation of sequence in the peptide binding groove; nevertheless, selective inhibitors of yeast and fungal NMTs have been discovered that bind in this location, exploiting residue differences at a handful of positions and often subtle differences in structure.^33,37^

The consensus sequence for N-myristoylation is loose and restrictive only over four of the six N-terminal residues.^38,39^ The N-terminal residue must be glycine, while at the second position, charged residues, proline and large hydrophobic residues are not allowed. At positions three and four, most, if not all, residues are allowed. Small but uncharged residues are allowed at position five (Ala, Ser, Thr, Cys, Asn and Gly) where serine is favoured, while the sole restriction at position six is that proline is not present (PROSITE pattern PS00008).^40^

**Fig. 6:**
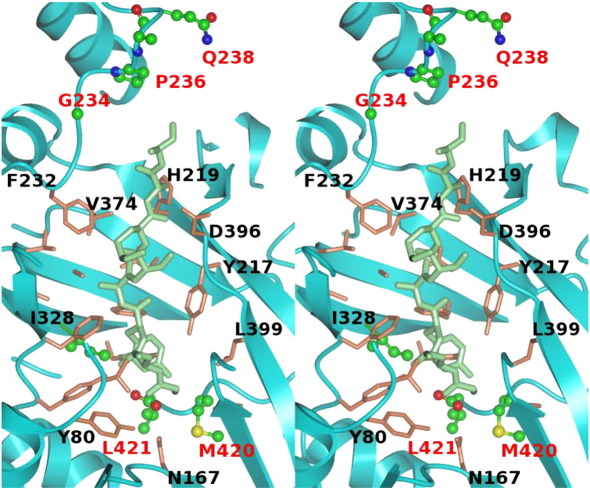
Conservation of residues in the peptide binding pocket of LdNMT and HsNMT. Stereo view of the GLYASKLA peptide from the ternary ScNMT complex (1iid.pdb) displayed in the context of the LdNMT peptide binding groove. The LdNMT structure (displayed as a cyan ribbon) was superposed onto that of the yeast NMT using the SSM routine in CCP4MG and is displayed together with the GLYASKLA peptide (thick light green cylinders) from ScNMT. The peptide runs N to C, bottom to top in the figure. Side chains of LdNMT residues in the vicinity of the peptide are displayed in thin cylinder format and coloured in coral for residues that are conserved in HsNMT and in ball-and-stick format with atoms coloured by element for positions where the LdNMT and HsNMT sequences diverge. Selected residues are labelled in black at conserved positions and red at divergent positions. The NHM species has been omitted for clarity.

To illuminate the character of the peptide binding surface in LdNMT, we superposed the binary complex of LdNMT with NHM onto the ternary complex of ScNMT with NHM and the octapeptide GLYASKLA (Fig. 4d). The octapeptide lies across the β-sheet in the C-terminal domain of the protein forming interactions with residues of strands k, l, m and g, loops DE, In, and Ab as well as with the C-terminal carboxylate and the fatty acyl-CoA (Fig. 4d). The residues that are predicted to surround this peptide in LdNMT are Tyr80, Val 81, Glu82, Phe88, Phe90, Tyr92, Asn167, Thr203, Tyr217, His219, Phe232, Tyr326, Ile328, Ser330, Leu341, Ala343, Tyr345, Val374, Asn376, Asp396, Leu399, Met420 and Leu421 (Fig. 6). These residues are conserved in LdNMT and HsNMT with the exception of Ile328 (Leu), Met420 (Leu) and Leu421 (Gln) (Figs. 3 and 6).

A high-resolution structure of HsNMT in complex with myristoyl-CoA has recently been released to the PDB (PDB code 3IU1; unpublished data) by the Structural Genomics Consortium (SGC). This structure and that of the binary complex of LdNMT can be superposed to give an rmsΔ of 1.25 Å in the positions of 369 equivalent C^α^ atoms. In relation to substrate binding, the superposition reveals differences in the loops forming the upper surface of the peptide binding site as viewed in Fig. 4, including the ordering of the Ab and kl loops, which are disordered in LdNMT. In contrast, there is disorder in the central residues of the DE loop in HsNMT. This loop is five residues longer in LdNMT (Fig. 3) and it is ordered in the parasite enzyme. The trajectory of the DE loop is altered so that it more closely surrounds the peptide binding pocket in HsNMT. It is predicted, based on comparison with GLYASKLA bound to ScNMT, that residue His313 in HsNMT would interact with the peptide substrate. This residue corresponds to Gly234 in LdNMT, and this and further residue differences in this loop at positions 236 to 238 in LdNMT may present an opportunity for selective inhibitor elaboration (Figs. 3 and 6).

The structures of ternary complexes of HsNMT in complex with myristoyl-CoA and three different polycyclic inhibitor molecules have also recently been released by the SGC (PDB codes 3IU2, 3JTK and 3IWE; unpublished data). The inhibitors reside in the extended groove distal to the myristoyl-CoA binding site and overlapping with the peptide binding site in ScNMT. The inhibitors partially fill the volume occupied by the N-terminal and central residues of the peptide^35^ and, in two cases, extend into the enlarged pocket for peptide side chain 4. The inhibitors also explore additional volume not occupied by the peptide, which is of significance for inhibitor discovery. In relation to the NMTs of other kinetoplastids, the residues predicted to form the peptide binding groove in *L. donovani* NMT (Fig. 6) are fully conserved in the NMTs from the pathogenic *Leishmania* species *L. major*, *L. infantum* and *L. braziliensis* and different in the NMTs of *T. brucei* and *T. cruzi* at two (Ile328 replaced by Leu and Leu421 by Val) and four (Tyr326 replaced by Phe, Ile328 by Leu, Gly234 by Leu, and Gln238 by His) positions, respectively.

### Concluding remarks

The studies described here show that *NMT* is likely to be an essential gene in the *L. donovani* life cycle, thus genetically validating LdNMT as a suitable target for drug discovery for VL. The overproduction and purification of recombinant LdNMT, and the demonstration of peptide myristoylation activity in an SPA that is adaptable to large-scale parallel application, pave the way for high-throughput inhibitor screening. The determination of the crystal structure of the LdNMT–NHM complex establishes a foundation both for the structural analysis of inhibitor complexes and for structure-assisted drug discovery. The next step is to carry out inhibitor screening. Industrial antifungal drug discovery programmes targeting NMTs led to the identification of highly potent inhibitors with selective action. However, for these programmes, directed at broad-spectrum inhibitors against a range of fungal pathogens, species selectivity was undesirable. These experiences augur well for the search for parasite-directed NMT inhibitors where broad-spectrum activity is not a requirement.

## Materials and Methods

### Parasite culture, generation of transgenic lines and infectivity analysis

Promastigotes of *L. donovani* MHOM/ET/67/L28 (LV9 strain) were maintained at 26 °C in modified RPMI 1640 medium (Sigma-Aldrich) supplemented with 20% fetal calf serum (Invitrogen). For targeted deletion of the single-copy *L. donovani* NMT gene, plasmids were generated containing either a hygromycin resistance gene (*HYG*) or a puromycin resistance gene (*PAC*) flanked with DNA sequences immediately upstream and downstream of the NMT open reading frame (ORF). These constructs, designated LdonNMTKO-*HYG* and LdonNMTKO-*PAC*, respectively, were created by replacing the corresponding *L. major* sequences in pNMT-*HYG*.^7^ To generate LdonNMTKO-*HYG*, primers were designed from the *L. infantum* NMT flanking sequences (LinJ32_20070420_V3). A 748 bp fragment immediately upstream of the NMT ORF was amplified using a proofreading DNA polymerase (KOD, Merck, UK) from *L. donovani* genomic DNA with primers LinfNMT5′Uf (5′-actaagcttCACACACTGTGAGCCTTGG-3′) and LinfNMT5′Ur (5′-atagtcgacCAGTCGCCCCTACTTGAGTC-3′). *L. infantum* sequence is shown in upper case. The amplified product was digested with HindIII–SalI and used to replace the corresponding *L. major* sequence in pNMT-*HYG*. A 952 -bp fragment immediately downstream of the *L. donovani* NMT ORF was similarly amplified using primers LinfNMT3′Uf (5′-atacccgggTTTCACCCTCCTTCACGTTC-3′) and LinfNMT3′Ur (5′-cctagatctCTGCCGAGAAAAGAGGTCAT-3′). After digestion with SmaI–BglII, this was used to replace the 3′-flanking *L. major* sequence at the corresponding site. To generate LdonNMTKO-*PAC*, *PAC* was released from plasmid pNMT-*PAC*^7^ and used to replace *HYG* at the SpeI–BamHI site in LdonNMTKO-*HYG*.  Linear targeting regions (5 μg; produced by digesting LdonNMTKO-*HYG* and LdonNMTKO-*PAC* with HindIII–BglII) were purified and used sequentially to transfect 2 × 10^7^ mid-log phase *L. donovani* promastigotes by nucleofection using a Human T Cell Nucleofector kit (Amaxa). Plasmid pTEXNMT Neo^7^ was also transfected into single allele *HYG* replacement clones to create NMT-overexpressing parasites. Individual clones of all lines were selected and characterised as described.^7^ Genomic DNA was analysed by PCR using the following primers: Ufor, 5′-CATTCCGAGAAGGGAGGGAG-3′; Nfor, 5′-CTACGTCGAGGACGACGACA-3′; Nrev, 5′-GTGTCTCGAGCTACAACATCACCAAGGCAACCTGA-3′. Southern blotting was carried out with digoxigenin (DIG)-labeled probes and hybridization reagents (Roche) as per the manufacturer's protocols. DIG-5′U probe, 780 bp, synthesized using primers LinfNMT5′Uf and LinfNMT5′Ur; DIG-NMT ORF probe, ∼ 1 kb, synthesized using primers Nfor and Nrev.

Groups of five BALB/c mice were infected with 4 × 10^7^ *L. donovani* metacyclic promastigotes (either wild type or one of the +/ΔNMT::*HYG* clones) intravenously via the tail vein in 200 μl of RPMI 1640 (GIBCO, Paisley, UK) and parasite burdens in liver and spleen were measured at 28 days post-infection, as previously described.^25^ Statistical analyses were performed by paired Student’s *t*-test. Experiments were approved by the University of York animal procedures and ethics committee and performed under UK Home Office license.

### Cloning, expression and purification of *L. donovani* NMT

The *NMT* ORF was amplified from *L. donovani* genomic DNA by PCR using primers based on the sequence of *NMT* from the closely related *L. infantum* strain JPCM5.^28^ Primers NMT-Xho (5′-GTGTggatccCGCAATCCATCGAACTCTGACGCT-3′) and NRev (see above) contain BamHI and XhoI recognition sites, respectively, for cloning of the amplified fragment into pGEX-6P (GE Healthcare) in order to express the enzyme as a C-terminal fusion protein with glutathione *S*-transferase. One clone with silent mutations in codons Ile360 (ATT→ATC) and Pro413 (CCC→CCT) was used to amplify a fragment using primers NMT-Xho and NMT-Nde (5′-GTTGTTGTTcatatgTCTCGCAATCCATCGAACTCTGAC-3′). This was cloned into a modified pET28 vector to incorporate an N-terminal hexahistidine tag. Both fusion proteins can be cleaved with HRV 3C protease to release NMT.

The recombinant plasmid encoding a His-tagged LdNMT was introduced into *E. coli* BL21(DE3) pRareS, and expression was achieved by growth in an autoinduction medium^41^ with overnight growth at 18 °C. Cell lysates were prepared by sonication in a buffer containing protease inhibitors, bovine pancreatic DNaseI, and a nonionic surfactant, Triton X-100 [0.1% (v/v)]. The unclarified cell lysate was loaded directly onto an immobilised metal-affinity chromatography column (GE Healthcare 1 ml HisTrap FF Crude). Fractions containing LdNMT were identified, pooled and diluted 10-fold in 50 mM Tris–HCl (pH 8.5) before loading onto an anion-exchange column (GE Healthcare 1 ml HiTrap Q HP) and elution by a linear gradient of 50 mM–0.5 M NaCl in buffer. Gel filtration on a Superdex 75 16/60 column (GE Healthcare) in 20 mM Tris buffer (pH 8.5), 200 mM NaCl and 2 mM DTT was used as a final purification step. Protein was concentrated and stored at − 20 °C in 25% glycerol, 20 mM Tris–HCl (pH 7.5), 100 mM NaCl, 2 mM DTT, 2 mM EGTA [ethylene glycol bis(β-aminoethyl ether) *N*,*N*′-tetraacetic acid] and 2 mM EDTA (ethylenediaminetetraacetic acid). The protein yield was typically 5 mg per litre of cells. For crystallography, the N-terminal tag was removed by incubation with a His-tagged HRV 3C protease (Novagen, 10 U per milligram His_6_-NMT protein) for 16 h at room temperature, before removal of any uncut protein (approximately 50% of total), cleaved His-tag and protease by passage through a 1 ml HisTrap HP Ni-column followed by buffer exchange.

### Protein crystallisation and structure solution

LdNMT [8 mg/ml in 50 mM Tris (pH 8.0), 50 mM NaCl] was crystallised by the vapour-diffusion method. Hanging drops consisting of 1.25 μl protein and 1.25 μl of reservoir solution containing 0.6 M lithium chloride, 20% (w/v) PEG (polyethylene glycol) 6000, 4% (v/v) 1,4-butanediol in 0.5 M sodium citrate buffer (pH 4.0) were equilibrated against 0.8 ml of reservoir solution at 20 °C. These conditions were derived from the JCSG+Suite (Qiagen) condition C2. Crystals appeared after a week with dimensions of less than 100 μm. Preliminary X-ray analysis established that the crystals belong to the orthorhombic space group *P*2_1_2_1_2_1_ with cell dimensions *a* =  46.0 Å, *b* =  90.1 Å, *c* =  92.4 Å. Assuming one molecule per asymmetric unit, the Matthews coefficient is 1.96 Å^3^/Da and the solvent content is 37%. Crystals were flash-vitrified directly in liquid nitrogen before storage.

Data were collected on synchrotron beamline ID23.1 (λ =  0.976 Å) at the European Synchrotron Radiation Facility (ESRF) (Grenoble) and processed using DENZO and SCALEPACK.^42^ Data collection and refinement statistics are summarised in Table 3. All data were used in molecular replacement calculations in the program MOLREP^43^ using the CaNMT coordinate set (PDB code 1NMT,^32^ 43% sequence identity) as the search model after making mutations to match the LdNMT sequence in the program CHAINSAW.^44^ Automated model building was carried out using flex-wARP,^45^ which found 384 residues with a sequence coverage of 90%. During this process the *R*_cryst_ dropped from 0.30 (*R*_free_ 0.32) to 0.21 (0.26). The model was refined by iterative cycles of REFMAC5^46^ using anisotropic temperature factors and TLS parameters, interspersed with manual modeling and adjustments carried out in COOT.^47^ Refinement was concluded with an *R*_cryst_ of 14.6% (*R*_free_ 19.1%) for data in the range 40–1.42 Å. The final model contains 64 cofactor NHM atoms, 378 water molecules and 402 protein residues, of which 91.5% lie in the most favoured, 8.2% in the additionally allowed and a single residue (Leu381) in a generously allowed region of the Ramachandran plot.

### Ligand binding and acyl transferase activity assay

Enzyme activity was measured by an SPA.^5^ The peptide substrate GLYVSRLFNRLFQKK(Biotin)-NH_2_ was stored as a 20 mM stock solution in dimethyl sulfoxide. Both [9,10(n)-^3^H]myristoyl-CoA [3.51 μM at 57 Ci/mmol in 0.01 M sodium acetate (pH 5.6)/ethanol (1:1) solution] and streptavidin PVT beads were purchased from GE Healthcare. A typical assay (100 μl scale) contained variable amounts of an inhibitor, 29.0 ng of purified LdNMT, 62.5 nM [^3^H]myristoyl-CoA (8 Ci/mmol) and 250 nM of peptide substrate in 4% dimethyl sulfoxide buffer 1 [30 mM Tris–HCl (pH 7.4), 0.5 mM EDTA, 0.5 mM EGTA, 2.5 mM DTT, 0.1% Triton X-100]. Incubation for 30 min at 37 °C was followed by the addition of 100 μl of bead-stop solution [1 mg/ml in 0.2 M phosphoric acid (pH 4.0), 0.75 mM MgCl_2_, 0.05% (w/v) bovine serum albumin, 0.01% (w/v) NaN_3_] to terminate the reaction. Overnight settling of the beads resulted in an accurate reading with high signal-to-noise ratio. The instrument used for counting was a Plate Chameleon multilabel reader (Hidex Oy, Finland).

The inhibitory activity of the non-hydrolysable myristoyl-CoA analogue (NHM) was measured at concentrations between 10 μM and 0.01 nM. The readout of [^3^H]-myristoylated peptide in the reaction lacking inhibitor was defined as 100% activity, and that from the reaction lacking enzyme was defined as 0%. The effects of the inhibitor at each concentration were calculated as a percentage of the control and the 50% inhibitory concentration (IC_50_) of the inhibitor was calculated by nonlinear regression analysis using Grafit 6.0.3 version (Erithacus Software Limited, UK). Assays were performed in triplicate.

The *K*_m_ for myristoyl-CoA was evaluated by varying the concentration of [^3^H]myristoyl-CoA between 500  and 0.23 nM, at a fixed 500 nM concentration of the assay peptide. The *K*_m_ with respect to peptide was evaluated by varying its concentration over the range 10–0.04 μM, at a fixed concentration of [^3^H]myristoyl-CoA of 62.5 nM. The amount of beads added in the stop solution was proportional to the amount of peptide used in the assay. The reactions were quenched after 1, 3 and 5 min, and the initial reaction velocities were derived. Data were analyzed by nonlinear regression (Michaelis–Menten) and linear regression of a double-reciprocal plot (Lineweaver–Burk) using Grafit 6.0.3 version (Erithacus Software Limited, UK). Assays were performed in duplicate.

### Synthesis of *S*-(2-oxo)pentadecyl-CoA

The non-hydrolysable myristoyl-CoA analogue (NHM) *S*-(2-oxo)pentadecyl-CoA was synthesised from commercially available pentadecan-2-one. In brief, the starting ketone was reacted with Hünig's base (diisopropylethylamine) and trimethylsilyl trifluoromethanesulfonate to form the terminal silyl enol ether, which was immediately reacted with *N*-bromosuccinimide to yield 1-bromopentadecan-2-one in 67% yield after purification by flash silica column chromatography. Direct displacement of the bromine with CoASH yielded, after purification by reversed-phase HPLC, *S*-(2-oxo)pentadecyl-CoA in 31% unoptimised yield. A full description of the synthesis is given in the Supplementary Material.

### Accession numbers

The nucleotide sequence of the gene encoding LdNMT (strain LV9) has been deposited in the EMBL DNA database with accession code FN555136. Coordinates and structure factors have been deposited in the PDB with accession number 2WUU.

## Figures and Tables

**Table 1:** Summary of *NMT* gene analyses of transgenic parasites

Clone properties	Method of analysis	+/ΔNMT::*HYG* + *PAC*	+/ΔNMT::*HYG* [pTEX NEO NMT] + *PAC*
Expressed NMT	Immunoblot	36/36	12/12 (H4 series)
NMT ORF present	PCR (primers Nfor + Nrev)	36/36	36/36
Maintained wild-type NMT locus	PCR (primers Ufor + Nrev)	33/36	2/36
Maintained wild-type NMT locus	DNA blot (NMT ORF probe)	34/35	1/12 (H4 series)^a^
*HYG*-replaced NMT locus	DNA blot (5′U probe)	28/35	36/36
*PAC*-replaced NMT locus	DNA blot (5′U probe)	18/35	36/36

a Multiple bands complicated analysis of H1 and H16 series.

**Table 2:** Kinetic and inhibition analysis

Enzyme	Myristoyl-CoA		Peptide		IC_50_ of non-hydrolysable myristoyl-CoA (nM)
	*V*_max_ (cpm/min)	*K*_m_^app^ (nM)	*V*_max_ (cpm/min)	*K*_m_^app^ (μM)	
LdNMT	243.0 ± 9.9	17.5 ± 2.9	230.4 ± 26.6	0.23 ± 0.12	68.7 ± 4.2
CaNMT^29^	713 ± 23	2.8 ± 0.4	831 ± 25	0.36 ± 0.04	nd
ScNMT^30^	nd	1.4	nd	0.9	nd

Peptide used with each enzyme: LdNMT, GLYVSRLFNRLFQKK(Biotin); CaNMT, GLTISKLFRR; ScNMT, GAAPSKIV-NH2. nd, not determined.

**Table 3:** Data collection and refinement statistics

*Data collection*	
Space group	*P*2_1_2_1_2_1_
Unit cell dimensions	
* a*, *b*, *c* (Å)	45.97, 90.13, 92.35
Resolution (Å)	40–1.42 (1.46–1.42)^a^
*R*_sym_^b^	0.097 (0.358)
〈*I*/σ(*I*)〉	10.8 (3.0)
Completeness (%)	98.1 (78.8)
Redundancy	7.4 (2.4)
	
*Refinement*	
Resolution (Å)	40.0–1.42
No. reflections	68,150 (3,986)
*R*_cryst_^c^ /*R*_free_^d^ (%)	14.6/19.1
No. atoms	
Protein	3446
Ligand	64
Water	378
*B*-factors (Å^2^)	
Protein	16.5
Ligand	14.3
Water	25.8
rmsd^e^	
Bond lengths (Å)	0.028
Bond angles (°)	2.18
Ramachandran plot^f^	91.5/8.2/0.3/0.0

a Values in parentheses are for the highest-resolution shell.

b *R*_sym_ = ∑_*hkl*_∑_*i*_|*I*_*i*_ − 〈*I*〉|/∑_*hkl*_∑_*i*_〈*I*〉 where *I*_*i*_ is the intensity of the *i*th measurement of a reflection with indexes *hkl* and 〈*I*〉 is the statistically weighted average reflection intensity.

c *R*_cryst_ = ∑||*F*_o_| − |*F*_c_||/∑|*F*_o_| where *F*_o_ and *F*_c_ are the observed and calculated structure factor amplitudes, respectively.

d *R*_free_ is the *R*_cryst_ calculated with 5% of the reflections chosen at random and omitted from refinement.

e Root-mean-square deviation of bond lengths or bond angles from ideal geometry.

f Percentage of residues in the most favoured/additionally allowed/generously allowed/disallowed regions of the Ramachandran plot.
