# Supplementary material for: N-Myristoyltransferase from Leishmania donovani: Structural and Functional Characterisation of a Potential Drug Target for Visceral Leishmaniasis
Source: J Mol Biol. 2010 Mar 5;396(4):985–99. doi: 10.1016/j.jmb.2009.12.032 (PMC2829124; doi:10.1016/j.jmb.2009.12.032)
Supplement: Supplementary material [file mmc1.doc]

**Supplementary Material.** Brannigan *et al.*, *N*-myristoyltransferase from *Leishmania donovani*: Structural and functional characterisation of a potential drug target for visceral leishmaniasis

**Synthesis of *S*-(2-oxo)pentadecyl CoA**

The non-hydrolysable myristoylCoA analogue (NHM) *S*-(2-oxo)pentadecyl CoA was synthesised (Figure S1) from commercially available pentadecan-2-one. In brief, the starting ketone was reacted with Hünig’s base (diisopropylethylamine, DiPEA) and trimethylsilyl trifluoromethanesulfonate (TMSOTf) to form the terminal silyl enol ether, which was immediately reacted with *N*-bromosuccinimide (NBS) to yield 1-bromopentadecan-2-one in 67% yield after purification by flash silica column chromatography. Direct displacement of the bromine with CoASH yielded, after purification by RP-HPLC, *S*-(2-oxo)pentadecyl CoA in 31% unoptimised yield.

All reagents and solvents, purchased from Sigma-Aldrich or Merck, were of the highest grade available, and used without further purification. NMR spectra were recorded in 5 mm tubes calibrated to the residual solvent peak stated, on Bruker AV-400 or 500 spectrometers. Electrospray ionisation (ESI) mass spectrometry was carried out on an Autospec P673 spectrometer. Ultrapure water was obtained from Elix® and MilliQ® Millipore water purification systems. Analytical and semi-preparative RP-HPLC was carried out on a Gilson system equipped with an autosampler. All non-aqueous reactions were carried out in oven-dried glassware under an atmosphere of dry nitrogen, with continuous stirring *via* a magnetic stirrer.

Pentadecan-2-one (0.5 g, 2.2 mmol) was dissolved in dichloromethane (10 ml), and cooled to 0 °C in an ice bath. Diisopropylethylamine (0.54 ml, 3.1 mmol) was added, followed by trimethylsilyl trifluoromethanesulfonate (0.49 ml, 2.7 mmol) dropwise, and the reaction stirred for 30 minutes at 0 °C to allow formation of the intermediate silyl enol ether. Sodium carbonate (0.47 g, 4.4 mmol) was then added, followed by *N*-bromosuccinimide (0.48 g, 2.7 mmol). The reaction was allowed to warm to ambient temperature and stirred for 2 hours before saturated sodium bicarbonate was added (10 ml). The organic phase was separated, washed with water (10 ml), brine (10 ml), dried over MgSO_4_, filtered and then evaporated to dryness *in vacuo*. The resulting yellow oil was purified by flash silica column chromatography (EtOAc/hexane, 0 to 10%) to yield 1-bromopentadecan-2-one as a slightly yellow oil (0.45 g, 67%). δ_H_ (400MHz; CDCl_3_): 3.87 (2H, s), 2.63 (2H, t, *J* 7.7), 1.64-1.55 (2H, m), 1.34-1.16 (20H, m), 0.86 (3H, t, *J* 7.0); HRMS (FAB, positive mode) found [MH]^+^ 304.1399 (C_15_H_30_BrO requires 304.1402).

Coenzyme A (0.10 g, 0.12 mmol) was dissolved in aqueous sodium carbonate (40 mM, 10 ml), DL-dithiothreitol (1.8 mg, 0.01 mmol) was added, and the mixture stirred at ambient temperature for 30 minutes. A solution of 1-bromopentadecan-2-one (0.183 g, 0.6 mmol) in *tert*-butanol (20 ml) was added, and the reaction mixture stirred for 16 hr. The volatile solvent was removed *in vacuo* (caution: mixture foams rapidly on evaporation) and the residue filtered over celite to remove excess 1-bromopentadecan-2-one. The aqueous solution was lyophilised to dryness, dissolved in sodium phosphate buffer (10 mM, pH 7.4) and purified by preparative reverse phase HPLC (C18 silica, 10 mM sodium phosphate, pH 7.4, 0% to 50% gradient of MeCN over 20 min), monitoring CoA absorbance at 258 nm. The majority of the residual CoA eluted as a broad peak at R_t_ = 14 min, followed by *S*-(2-oxo)pentadecyl CoA at R_t_ = 20 min, contaminated by residual CoA. The fractions containing the target compound were pooled, lyophilised and redissolved in water (1 ml). 5% aqueous perchloric acid was added (2 ml), whereupon a white precipitate formed. This white solid was isolated by centrifugation, washed with 1% perchloric acid (1 ml), acetone (1 ml), and dried over silica in a vacuum dessicator to yield *S*-(2-oxo)pentadecyl CoA (sodium salt) as a white fluffy solid (48 mg, 31%), >95% purity as judged by nuclear magnetic resonance spectroscopy and analytical RP-HPLC. δ_H_ (400MHz; d_6_-DMSO): 8.69 (1H, s), 8.43 (1H, s), 8.05 (1H, t, *J* 5.5), 7.76 (1H, t, *J* 6.1), 6.0 (1H, d, *J* 5.9), 4.79 (2H, br qn, *J* 4.1), 4.75-4.69 (2H, m), 4.45-4.39 (2H, m), 4.23-4.16 (2H, m), 3.91 (1H, dd, *J* 8.8, 5.2), 3.74 (1H, br s), 3.56 (1H, dd, *J* 8.8, 5.2), 3.40 (2H, s), 3.37-3.14 (4H, m), 2.26 (2H, t, *J* 7.1), 1.51-1.39 (2H. m), 1.31-1.16 (20H, m), 0.93 (3H, s), 0.85 (3H, t, *J* 7.0), 0.75 (3H, s); HRMS (ESI, negative mode) found [MNa_3_ – 4H]^-^ 1070.2798 (C_37_H_62_N_7_Na_3_O_17_P_3_S requires 1070.2840).


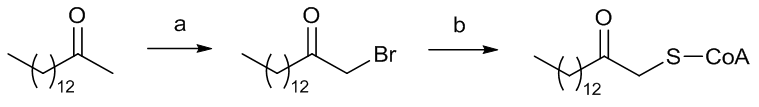


**Figure S1.** Synthesis of *S*-(2-oxo)pentadecyl CoA. a) i) DiPEA (1.4 eq), TMSOTf (1.2 eq), 0 °C, 30 min; ii) Na_2_CO_3_ (2 eq), NBS (1.2 eq), r.t., 2h; b) CoASH, DTT (0.1 eq), Na_2_CO_3(aq)_/*^t^*BuOH 1:2, 16h.

**Inhibition of LdNMT**

**by non-hydrolysable Myr-CoA**

IC_50_ = 68.7 ± 4.2 nM (slope factor 0.82)

**Km of Myr-CoA for LdNMT**

Km^app^ = 17.5 ± 2.9 nM

Vmax = 243.0 ± 9.9 cpm/min

**Km of peptide for LdNMT**

Km^app^ = 0.23 ± 0.12 μM

Vmax = 230.4 ± 26.6 cpm/min

**Figure S2. Kinetic and inhibition analysis for *L. donovani* NMT**
